# Cross‐cultural validation of plasma p‐tau217 and p‐tau181 as precision biomarkers for amyloid PET positivity: An East Asian study in Taiwan and Korea

**DOI:** 10.1002/alz.14565

**Published:** 2025-01-29

**Authors:** Yung‐Shuan Lin, Hyuk Sung Kwon, Wei‐Ju Lee, Mina Hwang, Jee Hyang Jeong, Seong‐Ho Koh, Seong Hye Choi, Jong‐Ling Fuh

**Affiliations:** ^1^ Department of Neurology, Neurological Institute Taipei Veterans General Hospital Taipei Taiwan; ^2^ School of Medicine, College of Medicine National Yang Ming Chiao Tung University Taipei Taiwan; ^3^ Brain Research Center National Yang Ming Chiao Tung University Taipei Taiwan; ^4^ Department of Neurology Hanyang University Guri Hospital Hanyang University College of Medicine Guri Republic of Korea; ^5^ Neurological Institute Taichung Veterans General Hospital Taichung Taiwan; ^6^ Department of Post‐Baccalaureate Medicine, College of Medicine National Chung Hsing University Taichung Taiwan; ^7^ Department of Neurology Ewha Woman's University College of Medicine Seoul Republic of Korea; ^8^ Department of Neurology Inha University College of Medicine Incheon Republic of Korea

**Keywords:** Alzheimer's disease, amyloid positron emission tomography, apolipoprotein E, glial fibrillary acidic protein, phosphorylated tau181, phosphorylated tau217

## Abstract

**INTRODUCTION:**

Plasma phosphorylated tau (p‐tau) biomarkers have improved Alzheimer's disease (AD) diagnosis, but data from diverse Asian populations are limited. This study evaluated plasma p‐tau217 and p‐tau181 levels in Korean and Taiwanese populations.

**METHODS:**

All participants (*n* = 270) underwent amyloid positron emission tomography (PET) and blood tests. Plasma p‐tau model‐derived probabilities of amyloid PET positivity (amyloid beta [Aβ]+) classified participants into low‐, intermediate‐, or high‐risk groups.

**RESULTS:**

In both cohorts, plasma p‐tau217 outperformed p‐tau181, especially in cognitively unimpaired participants (area under the curve = 0.921 [p‐tau217] vs. 0.769 [p‐tau181], *P*_difference _= 0.022). Including apolipoprotein E status and glial fibrillary acidic protein improved model fit. The negative predictive value of the low‐risk group and positive predictive value of the high‐risk group were 97.5% and 86.0%, respectively.

**DISCUSSION:**

Plasma p‐tau217 and p‐tau181 effectively predict Aβ+ among culturally different Asian populations. P‐tau217 performed better, especially in the early stages of AD. Plasma p‐tau217–based models reduced intermediate‐risk classifications, suggesting fewer amyloid PET scans needed to confirm the diagnosis.

**Highlights:**

- The efficacy of plasma phosphorylated tau (p‐tau)217 and p‐tau181 was analyzed in two Asian populations.
- Plasma p‐tau217 performs better in predicting amyloid positron emission tomography positivity, especially in cognitively unimpaired subjects.
- Adding apolipoprotein E and glial fibrillary acidic protein to p‐tau improved model accuracy.
- The models from each cohort were confirmed in the other cohort.
- Plasma p‐tau–based risk stratification may reduce the need for confirmatory tests.

## 1 BACKGROUND

The development of anti‐amyloid therapies and the increasing burden of Alzheimer's disease (AD) due to population aging^1^, ^2^, ^3^ has prioritized the early detection of individuals with positive amyloid beta (Aβ+) pathology. Cerebrospinal fluid (CSF) or amyloid positron emission tomography (PET) are used in clinical practice to identify the Aβ+ accurately but have limitations due to their high cost or invasiveness.^4^ Given these constraints, screening many individuals would be challenging. To overcome these limitations, the importance of blood biomarkers has been emphasized, and numerous studies have been conducted.^5^, ^6^

Among blood biomarkers, plasma phosphorylated tau (p‐tau) biomarkers, including p‐tau217, p‐tau181, and p‐tau231, differ in phosphorylation sites and have garnered significant attention for their ability to predict Aβ+. In the revised criteria for diagnosing AD by the Alzheimer's Association (AA) published in 2024, plasma p‐tau217, p‐tau181, and p‐tau231 are considered biomarkers that become abnormal simultaneously as abnormal amyloid PET imaging.^3^ P‐tau181 is strongly associated with CSF p‐tau181^7^ and predicts amyloid positivity and dementia progression, although it becomes abnormal at relatively higher amyloid burdens.^8^ By contrast, plasma p‐tau217 outperforms other p‐tau species and has demonstrates diagnostic performance equivalent to that of CSF p‐tau217 in predicting Aβ+.^7^, ^9^ Meanwhile, p‐tau231 is the earliest biomarker to surpass abnormal thresholds, becoming elevated at lower amyloid PET levels (26.4 Centiloids), compared to p‐tau217 and p‐tau181.^10^ These differences highlight their complementary roles in reflecting various stages of AD pathology, with p‐tau231 identifying very early changes, p‐tau217 excelling in amyloid differentiation, and p‐tau181 capturing later tau‐related processes. While plasma p‐tau217 shows potential as a diagnostic tool for AD, the limited availability of commercial tests has hindered its widespread use.^11^ Furthermore, some studies have highlighted racial disparities in plasma biomarkers for AD, and there is a lack of data regarding ethnic variations in Asian populations.^12^, ^13^ Cultural differences may affect plasma p‐tau levels and cut‐off values, and variations in accuracy could be influenced by different laboratory settings. In addition, apolipoprotein E (*APOE*) ε4 carriers have a two to three times higher prevalence of amyloid pathology and exhibit earlier and more significant increases in plasma p‐tau181 levels than non‐carriers.^14^, ^15^ *APOE* ε4 modifies the relationship between Aβ and tau pathologies, such that individuals with ε4 alleles exhibit higher CSF p‐tau levels at equivalent brain amyloid burdens.^16^ Similarly, astrocyte reactivity, indicated by elevated glial fibrillary acidic protein (GFAP), critically modulates Aβ’s effects on tau phosphorylation,^17^ with tau pathology accumulating in proportion to amyloid burden only in individuals with astrocyte reactivity.

The accurate prediction of amyloid pathology using plasma p‐tau alone is challenging in the clinical setting because of the complexity and heterogeneity of AD. We hypothesized that combining plasma p‐tau with *APOE* genotype and GFAP levels would enhance the prediction of amyloid positivity. Hence, we evaluated plasma p‐tau217 and p‐tau181, both individually and in combination with other biomarkers including GFAP and *APOE*. This study focused on cohorts from two Asian countries with distinct cultural backgrounds, addressing an area that has not been explored previously. Plasma p‐tau217 and p‐tau181 were measured using the commercially available Simoa immunoassay. The p‐tau–based models from each cohort were validated in another cohort. P‐tau–based models were used to stratify participants into low‐, intermediate‐, or high‐risk amyloid PET positivity groups, demonstrating that this risk stratification can reduce the number of patients requiring confirmatory tests.

## 2 METHODS

### 2.1 Study participants

We analyzed participants from two independent cohorts: the Korea Brain Aging Study for the Early Diagnosis and Prediction of Alzheimer's Disease (KBASE‐V, Korean cohort) and the AD biosignature study (Taiwanese cohort). One hundred seventy individuals (93, 41, and 36 patients who were cognitively unimpaired [CU], had mild cognitive impairment [MCI], and had clinically probable AD dementia, respectively) underwent both amyloid PET and blood biomarker measurements. The detailed methods, including the inclusion and exclusion criteria of the KBASE‐V have been described elsewhere.^18^ The KBASE‐V study was approved by the institutional review board (IRB) of each participating center (INHAUH 2015‐03‐021), comprising a nationwide cohort of participants from nine hospitals. All CU participants had education‐adjusted performance within the normal range on memory tests and a Clinical Dementia Rating (CDR) scale score of 0 points. The participants with MCI met the following criteria proposed by Petersen et al.^19^: (1) a CDR score of 0.5 points; (2) memory complaint, preferably corroborated by an informant; (3) impaired memory function for age and education; (4) preserved general cognitive function; (5) intact activities of daily living; and (6) not demented. Participants with AD met (1) the diagnostic criteria for dementia, as per the Diagnostic and Statistical Manual of Mental Disorders 4th Edition (DSM‐IV‐TR) and (2) the criteria for probable AD as per the National Institute of Neurological and Communicative Disorders and Stroke–Alzheimer's Disease and Related Disorders Association (NINCDS‐ADRDA) criteria.^20^ All participants in the KBASE‐V were aged 55 to 90 years and underwent physical and neurological examinations, including the Mini‐Mental State Examination (MMSE),^21^ the CDR scale,^22^ and the Consortium to Establish a Registry for Alzheimer's Disease,^23^ yearly. All participants underwent 3.0 T brain magnetic resonance imaging (MRI).

Participants in the Taiwanese cohort were prospectively enrolled from the memory clinics of the Taipei Veterans General Hospital (VGH) and Taichung VGH, as described previously.^24^ CU participants were volunteers with normal cognitive function. MCI was diagnosed according to the revised consensus criteria by Petersen et al.^19^ The cutoff value for diagnosing MCI was set at 1.5 standard deviations below the age‐adjusted norm for the Wechsler Memory Scale III logical memory test. AD dementia diagnoses required multidisciplinary consensus, according to the clinical criteria for probable AD as described by the National Institute on Aging–AA (NIA‐AA) in 2011.^25^ The exclusion criteria included patients with significant neurological conditions other than AD. These conditions included acute confusion due to systemic disease, abnormality, or drug intoxication; major depressive disorder according to the DSM‐V; probable vascular dementia; normal pressure hydrocephalus; progressive supranuclear palsy; and a history of significant head trauma followed by persistent neurological deficits or known structural brain abnormalities. The study was approved by the IRBs of both the Taipei and Taichung VGHs (2012‐05‐033B; 2022‐01‐005ACF; SF12171) in compliance with the Declaration of Helsinki. All participants in the Taiwanese cohort underwent a standardized evaluation that included physical examination, clinical interviews, neuropsychological tests (MMSE, CDR, 12‐item memory test, 15‐item modified Boston Naming Test, and categorical verbal fluency), and brain MRI. One hundred participants (37, 29, and 34 patients with CU, MCI, and dementia, respectively) underwent amyloid PET imaging and blood biomarker testing.

**RESEARCH IN CONTEXT**

1. **Systematic review**: We reviewed the literature on the use of plasma phosphorylated tau (p‐tau) for detecting amyloid beta (Aβ) positivity. Previous reports have suggested that p‐tau217 is a promising diagnostic tool for Alzheimer's disease (AD). However, information on the efficacy of p‐tau217, measured by commercially available methods, in predicting Aβ positivity or reducing the number of confirmatory tests is lacking, especially in Asians with diverse cultural backgrounds.
2. **Interpretation**: Both plasma p‐tau217 and p‐tau181 effectively predicted amyloid PET positivity, with p‐tau217 showing superior performance, especially in the cognitively unimpaired stage. Adding apolipoprotein E and glial fibrillary acidic protein to p‐tau improved the model accuracy, and models were validated across cohorts. Compared to the p‐tau181–based models, the p‐tau217–based models resulted in fewer patients requiring confirmatory tests.
3. **Future directions**: Future research should verify the suitability of implementing plasma p‐tau–based detection methods in primary care settings. Efforts are needed to reduce p‐tau variability across different laboratory environments.

The criteria for CU and MCI were the same in both cohorts. Different clinical criteria for AD were used in the two cohorts (i.e., the DSM‐IV‐TR and NINCDS‐ADRDA criteria in the Korean cohort and the NIA‐AA 2011 clinical criteria in the Taiwanese cohort).

### 2.2 PET images

All participants were assigned to the Aβ+ or Aβ− groups according to the amyloid status on PET, irrespective of their cognitive status. Positive amyloid pathology by Aβ PET was defined as a cutoff point of 37 Centiloid units (according to the criteria of the TRAILBALZER trial).^2^ Centiloid replication analysis was performed in both cohorts as described previously.^26^

In the Korean cohort, all participants underwent Aβ PET (^11^C Pittsburgh compound B [PiB] PET or ^18^F‐flutemetamol PET) at baseline. The standardized uptake value ratio (SUVR) was obtained using the pons as the reference region for ^18^F‐flutemetamol PET and the cerebellar gray matter for ^11^C‐PiB PET.

All participants in the Taiwanese cohort underwent ^18^F‐florbetaben (FBB) amyloid PET at both the Taipei and Taichung VGHs*. *The participants underwent a dynamic PET emission scan in 3D mode with continuous brain PET data collected for 20 minutes. PET images were acquired 90 to 110 minutes postinjection (296 MBq). A freeware tool AmPQ (available at https://sites.google.com/view/cgu‐mipal/ampq) was used to convert FBB PET SUVR to the Centiloid scale.

### 2.3 Plasma sampling and analysis

Plasma p‐tau181, p‐tau217, and GFAP levels were analyzed using the same method in both cohorts. Plasma p‐tau181 levels were measured in all participants. P‐tau217 was analyzed in 80% of the Korean cohort (*n* = 136) and 75% of the Taiwanese cohort (*n* = 75). GFAP levels were measured in 94.1% of the Korean cohort (*n* = 160) and 98% of the Taiwanese cohort (*n* = 98).

Plasma samples from both countries were collected following the Alzheimer's Disease Neuroimaging Initiative 2 (ADNI‐2) protocol.^27^ Blood samples were collected after overnight fasting for at least 6 hours, centrifuged within an hour, and stored at –80°C. The plasma levels of p‐tau181 were quantified using the Simoa Human p‐Tau181 Advantage V2 assay (Quanterix PN/104111), the p‐tau217 plasma levels were assessed using the Simoa Human ALZpath p‐tau217 V2 assay (Quanterix, PN/104371), and the levels of plasma GFAP were measured using the GFAP Advantage PLUS assay (Quanterix, PN/102336).^11^ The standard protocol allocated plasma samples and controls into 96‐well Quanterix plates for duplicate assessments. The instrument eases the onboard dilution with 4× dilution for p‐tau181, 3× dilution for p‐tau217, and 2× dilution for GFAP. Following the manufacturer's protocol, the assay was performed using a two‐step digital immunoassay with the Simoa HD‐X Analyzer (Quanterix). The plasma samples were subjected to a single thawing and refreezing cycle before analyzing p‐tau. *Z* score transformations were applied to the plasma biomarkers in each cohort to facilitate comparisons between the Korean and Taiwanese cohorts.

The *APOE* genotype was identified by extracting genomic DNA from venous blood. Genomic DNA was isolated from whole blood using a Gentra Puregene kit (Qiagen), Wizard Genomic DNA Purification Kit (Promega) or ExiPrep16 Dx Kit (Bioneer), according to the manufacturer's protocol. The presence of the ε2, ε3, and ε4 alleles of the *APOE* gene was determined by genotyping of single nucleotide polymorphisms rs429358 and rs7412. An *APOE* ε4 carrier was defined as having at least one ε4 allele (including ε2/ε4, ε3/ε4, and ε4/ε4) and coded as a binary variable (1 for ε4 carriers and 0 for non‐carriers). rs429358 and rs7412 were genotyped using the TaqMan genotyping assay (Applied Biosystems) or AccuPower ApoE real‐time polymerase chain reaction (PCR) kit (Bioneer). PCR analysis was performed with 96‐well microplates using an ABI StepOnePlus real‐time PCR machine (Applied Biosystems), 7500 real‐time PCR machine (Applied Biosystems) or Exicycler 96 real‐time PCR system (Bioneer). Alleles were discriminated by detecting fluorescence using the StepOne software 2.3 (Applied Biosystems) or ExiGenotyper 1.01.4 (Bioneer).

### 2.4 Statistical analysis

Pearson chi‐square, Student's *t*‐test and Mann–Whitney *U* tests were used to compare baseline demographics, clinical data, and biomarker levels. To further compare the plasma biomarkers between the two cohorts, participants were matched for age, sex, cognitive stage, and amyloid positivity (*n* = 36 in each cohort). The discrimination accuracies of the plasma biomarkers were determined using receiver operating characteristic (ROC) curve analysis to correctly identify the amyloid status on PET. All the models were adjusted for age and sex. The areas under the curve (AUCs) of different ROC curves were compared using the DeLong method. Improvements in model fit were estimated using the Akaike information criterion (AIC), with a decrease of two or more in the AIC indicating a better model fit.^28^

We applied two thresholds (a two‐step workflow) derived from the plasma p‐tau model probabilities for amyloid PET positivity. These strategies are defined based on a lower probability threshold with 95% sensitivity and a higher probability threshold with 95% specificity. Participants with a probability below the lower threshold (95% sensitivity) were classified as low risk, whereas those with a higher threshold (95% specificity) were considered high risk. Individuals in between were categorized as having an intermediate risk. The negative predictive value (NPV) was evaluated as the prevalence of amyloid PET–negative cases in the low‐risk group. In contrast, the positive predictive value (PPV) was assessed as the prevalence of amyloid PET–positive cases in the high‐risk group. The number of scans saved was calculated as 100% minus the percentage in the intermediate‐risk group.

Statistical analyses were performed using IBM SPSS Statistics for Windows, version 27.0 (IBM Corp.) and R version 4.2.1 (R Foundation for Statistical Computing). Significance was defined as a two‐sided *P* value < 0.05.

## 3 RESULTS

### 3.1 Participant characteristics

The baseline characteristics and blood biomarker levels of both cohorts are presented in Table 1. In the Korean cohort, 43 participants (25.3%) were Aβ+. Specifically, 8 (8.6%) CU, 10 (24.4%) MCI, and 25 (69.4%) clinically probable AD dementia patients were Aβ+. Compared to participants who were Aβ−, those who were Aβ+ were older; had a lower body mass index; had greater rates of dementia and carrying *APOE* ε4; had lower MMSE and CDR scores; and had greater p‐tau217, p‐tau181, and GFAP levels. In the Taiwanese cohort, 2 (5.4%) CU, 8 (27.6%) MCI, and 20 (58.8%) clinically probable dementia were Aβ+. Participants who were Aβ+ had greater rates of dementia and a higher prevalence of carrying the *APOE* ε4 allele; had lower MMSE scores; and had greater levels of plasma p‐tau217, p‐tau181, and GFAP. Other demographics and the distributions of other risk factors were similar between the Aβ− and Aβ+ groups.

**TABLE 1:** Baseline characteristics and plasma biomarkers of patients according to amyloid positivity on PET in the Korean and Taiwanese cohorts.

	Combined cohort			Korean cohort			Taiwanese cohort		
	Aβ negative (*n* = 197)	Aβ positive (*n* = 73)	*p*	Aβ negative (*n* = 127)	Aβ positive (*n* = 43)	*p*	Aβ negative (*n* = 70)	Aβ positive (*n* = 30)	*p*
Demographics									
Age, years	68.9 ± 8.0	72.0 ± 9.0	0.007^a^	68.4 ± 8.0	74.0 ± 7.9	<0.001^a^	69.6 ± 8.0	69.0 ± 9.8	0.728^a^
Sex, females (%)	93 (47.2)	32 (43.8)	0.622	72 (56.7)	27 (62.8)	0.483	32 (45.7)	14 (46.7)	0.930
Education, years	10.8 ± 4.9	10.2 ± 4.7	0.356^a^	9.9 ± 5.1	8.3 ± 4.4	0.076^a^	12.6 ± 4.1	13.0 ± 3.7	0.665^a^
BMI, kg/m^2^	24.3 ± 3.0	23.0 ± 2.9	0.002^a^	24.3 ± 3.0	22.7 ± 2.6	<0.003^a^	24.4 ± 3.2	23.4 ± 3.2	0.172^a^
Cognitive stage			<0.001			<0.001			<0.001
CU (%)	120 (60.9)	10 (13.7)		85 (66.9)	8 (18.6)		35 (50.0)	2 (6.7)	
MCI (%)	52 (26.4)	18 (24.7)		31 (24.4)	10 (23.3)		21 (30.0)	8 (26.7)	
Dementia (%)	25 (12.7)	45 (61.6)		11 (8.7)	25 (58.1)		14 (20.0)	20 (66.7)	
Medical history									
Hypertension	80/195 (41.0)	28 (38.4)	0.692	53/125 (41.7)	19 (44.2)	0.838	27 (38.6)	9 (30.0)	0.413
Diabetes mellitus	33 (16.8)	8 (11.0)	0.239	21 (16.5)	6 (14.0)	0.689	12 (17.1)	2 (6.7)	0.166
Dyslipidemia	60/194 (30.9)	10 (13.7)	0.004	52/124 (41.9)	10 (23.3)	0.029	8 (41.1)	0 (0.0)	0.054
Coronary artery disease	10 (5.1)	4 (5.5)	0.894	6 (4.7)	3 (7.0)	0.189	4 (5.7)	1 (3.3)	0.617
Cerebrovascular disease	7 (3.6)	2 (2.7)	0.741	5 (3.9)	2 (4.7)	0.839	2 (2.9)	0 (0.0)	0.350
MMSE score, median (IQR)	27.0 (23.5–29.0)	21.0 (15.0–25.0)	<0.001^b^	26.0 (22.0–28.0)	20.0 (15.0–25.0)	<0.001^b^	27.0 (25.0–29.0)	21.5 (15.8–25.3)	<0.001^b^
CDR score, median	0.0 (0.0–0.5)	0.5 (0.5–1.0)	<0.001^b^	0.0 (0.0–0.5)	0.5 (0.5–1.0)	<0.001^b^	0.3 (0.0–0.5)	0.5 (0.5–1.0)	<0.001^b^
CDR‐SOB score, median	0.0 (0.0–1.0)	3.0 (0.8–5.8)	<0.001^b^	0.0 (0.0–0.5)	2.5 (0.5–5.0)	<0.001^b^	0.3 (0.0–2.1)	3.3 (1.4–7.0)	<0.001^b^
*APOE* ε4 carrier	31 (15.8)	40 (54.8)	<0.001	24 (18.9)	21 (48.8)	<0.001	7 (10.1)	19 (63.3)	<0.001
Plasma p‐tau217 (pg/mL)^c^	0.39 ± 0.42	1.35 ± 0.70	<0.001^a^	0.44 ± 0.46	1.58 ± 0.72	<0.001^a^	0.29 ± 0.33	1.00 ± 0.52	<0.001^a^
Plasma p‐tau181 (pg/mL)	2.11 ± 1.61	4.34 ± 2.16	<0.001^a^	2.29 ± 1.88	4.96 ± 2.41	<0.001^a^	1.79 ± 0.85	3.46 ± 1.34	<0.001^a^
Plasma GFAP (pg/mL)^c^	253.3 ± 170.1	543.7 ± 296.5	<0.001^a^	269.5 ± 159.4	641.8 ± 306.3	<0.001^a^	225.1 ± 185.0	408.3 ± 224.0	<0.001^a^

*Note*: Data are presented as mean ± standard deviation, median (IQR) or number (%) unless otherwise indicated. Cut‐off for amyloid PET positivity: Centiloid 37.

Pearson's Chi‐Square test was used to analyze the association of sex, cognitive stage, hypertension, diabetes mellitus (DM), dyslipidemia, coronary artery disease (CAD), cerebrovascular disease (CVD), and *APOE* ε4 carrier status.

Abbreviations: Aβ, amyloid beta; *APOE*, apolipoprotein E; BMI, body mass index; CDR, Clinical Dementia Rating; CDR‐SOB, Clinical Dementia Rating Scale Sum of Boxes; CU, cognitively unimpaired; GFAP, glia fibrillary acidic protein; IQR, interquartile range; MCI, mild cognitive impairment; MMSE, Mini‐Mental State Examination; PET, positron emission tomography; p‐tau, phosphorylated tau.

^a^ Student *t* test.

^b^ Mann–Whitney *U* test.

^c^ p‐tau217 was analyzed in 211 (78.1%) and GFAP was analyzed in 258 (95.6%).

**FIGURE 1:**
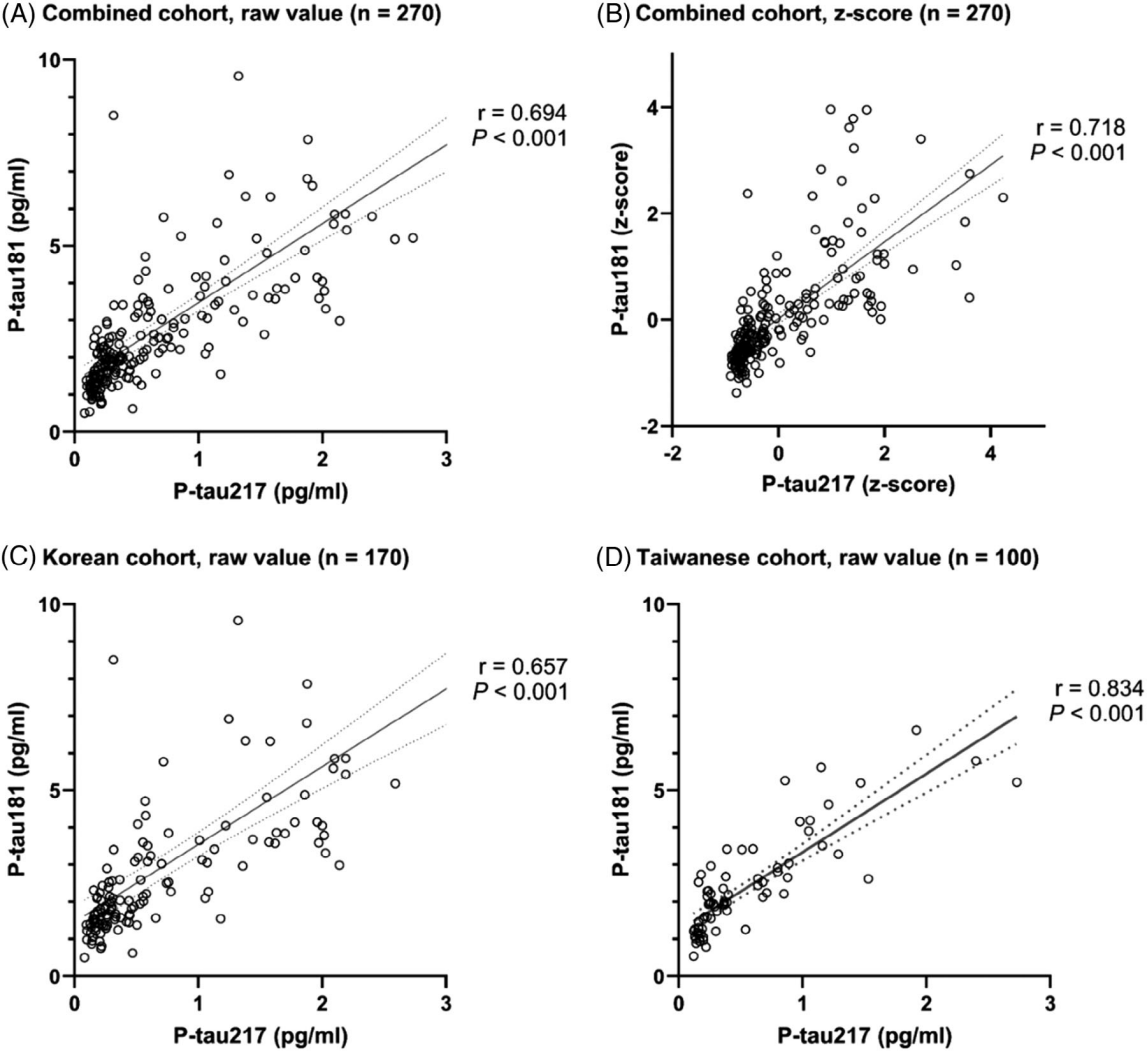
Correlation of plasma p‐tau217 and p‐tau181 levels in the combined, Korean, and Taiwanese cohorts. Raw values in the combined cohort (A), Korean cohort (C), and Taiwanese cohort (D). *Z* scores of the combined cohort (B). p‐tau, phosphorylated tau.

The plasma p‐tau217 level was significantly correlated with the p‐tau181 level (Figure 1), regardless of whether raw values or *z* scores were analyzed (*r* = 0.694, *P *< 0.001) or (*r* = 0.718, *P* value < 0.001).

### 3.2 Predicting amyloid positivity confirmed by PET

Among all the participants, univariate analysis revealed a significant association between amyloid PET positivity and age (odds ratio [OR] = 1.047, 95% confidence interval [CI] = 1.012–1.083), p‐tau217 (OR = 1.241, 95% CI = 1.172–1.313), p‐tau181 (OR = 1.155, 95% CI = 1.108–1.203), GFAP (OR = 1.163, 95% CI = 1.111–1.126), cognitive stage, hyperlipidemia (OR = 0.354, 95% CI = 0.170–0.738), *APOE* ε4 carriers (OR = 6.452, 95% CI = 3.524–11.752), and MMSE score (OR = 0.799, 95% CI = 0.750–0.851; Table 2). In the multivariable analysis, only increased plasma p‐tau217 (OR = 1.181, 95% CI = 1.077–10.889) and *APOE* ε4 status (OR = 4.052, 95% CI = 1.508–10.889) were independently associated with amyloid PET positivity (Table 2).

**TABLE 2:** Multivariable logistic regression analysis for amyloid positivity.

	Unadjusted OR	Adjusted OR model	*p* value
Age	1.047 (1.012–1.083)	1.013 (0.955–1.074)	0.674
Sex	0.873 (0.508–1.498)	1.106 (0.422–2.900)	0.837
Plasma p‐tau217 per 0.1 *z* score increase	1.241 (1.172–1.313)	1.181 (1.077–10.889)	<0.001
Plasma p‐tau181 per 0.1 *z* score increase	1.155 (1.108–1.203)	0.987 (0.930‐1.049)	0.679
Plasma GFAP per 0.1 *z* score increase	1.163 (1.111–1.216)	0.987 (0.930‐1.049)	0.681
Cognitive stage			
CU	Ref	Ref	0.414
MCI	4.154 (1.796‐9.609)	2.311 (0.658–8.113)	0.191
Dementia	21.60 (9.614‐48.527)	2.231 (0.423–11.768)	0.344
Hyperlipidemia	0.354 (0.170–0.738)	0.521 (0.157–1.732)	0.288
*APOE* ε4 carrier	6.452 (3.524–11.752)	4.052 (1.508‐10.889)	0.006
MMSE, per 1 score increase	0.799 (0.750–0.851)	0.968 (0.851–1.100)	0.615

*Note*: Data are presented as odds ratios (95% confidence intervals). *P* value for multivariate models. Adjusted variables were age, sex, and selected from the results of the unadjusted univariate analysis with *P *< 0.05: age, sex, plasma p‐tau217, plasma p‐tau181, plasma GFAP, cognitive stage, hyperlipidemia, *APOE* ε4 carrier, and MMSE score.

Abbreviations: *APOE*, apolipoprotein E; CU, cognitively unimpaired; GFAP, glial fibrillary acidic protein; MCI, mild cognitive impairment; MMSE, Mini‐Mental State Examination; OR, odds ratio; p‐tau, phosphorylated tau.

**FIGURE 2:**
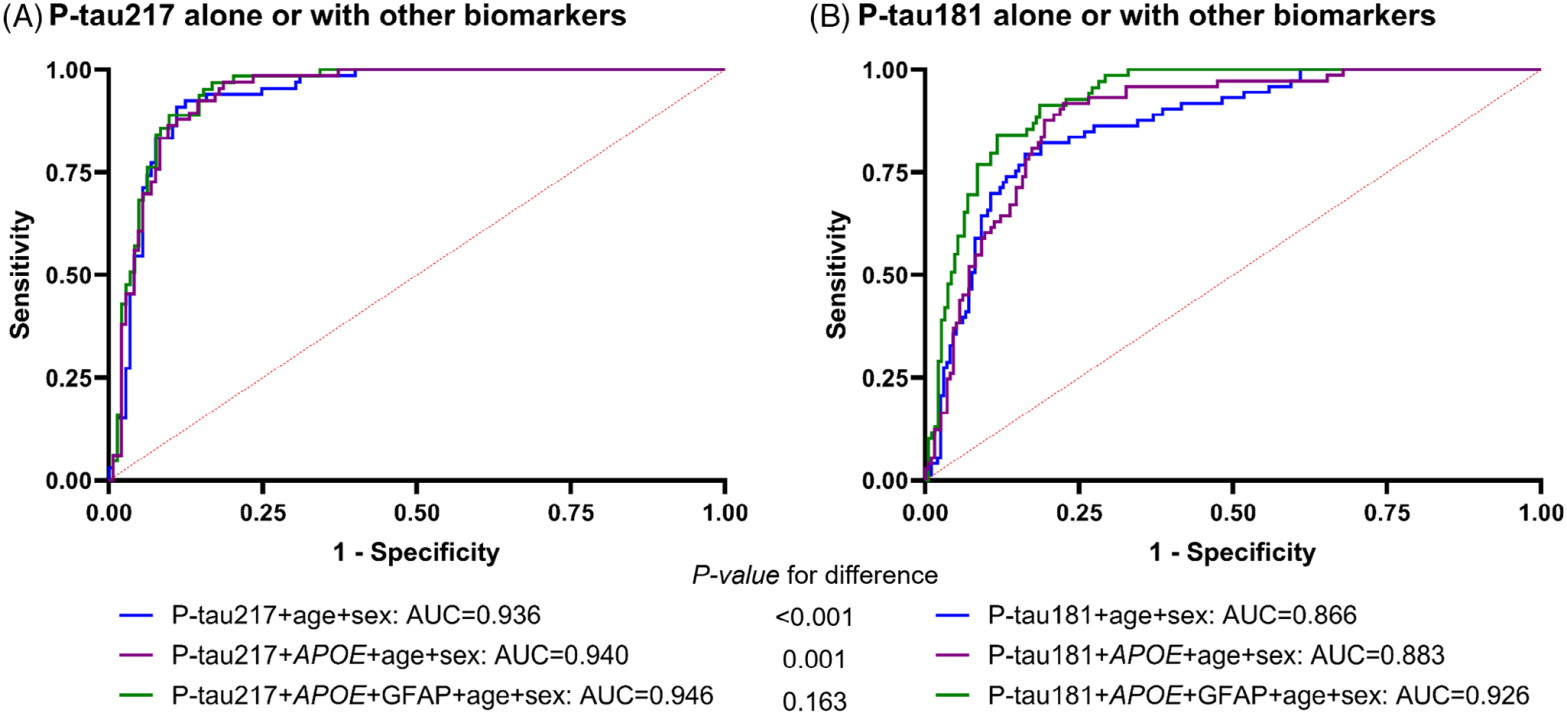
ROC curve using p‐tau alone or in combination with other biomarkers for predicting Aβ status using PET. ROC curve analysis of p‐tau217 (A) and p‐tau181 (B) alone or in combination with other biomarkers for predicting Aβ status in the combined cohorts. ROC curves are shown for plasma p‐tau; plasma p‐tau combined with *APOE* status; and the combination of plasma p‐tau, *APOE* status, and plasma GFAP. Aβ, amyloid beta; *APOE*, apolipoprotein E; AUC, area under the curve; GFAP, glial fibrillary acidic protein; PET, positron emission tomography; p‐tau, phosphorylated tau; ROC, receiver operating characteristic.

**FIGURE 3:**
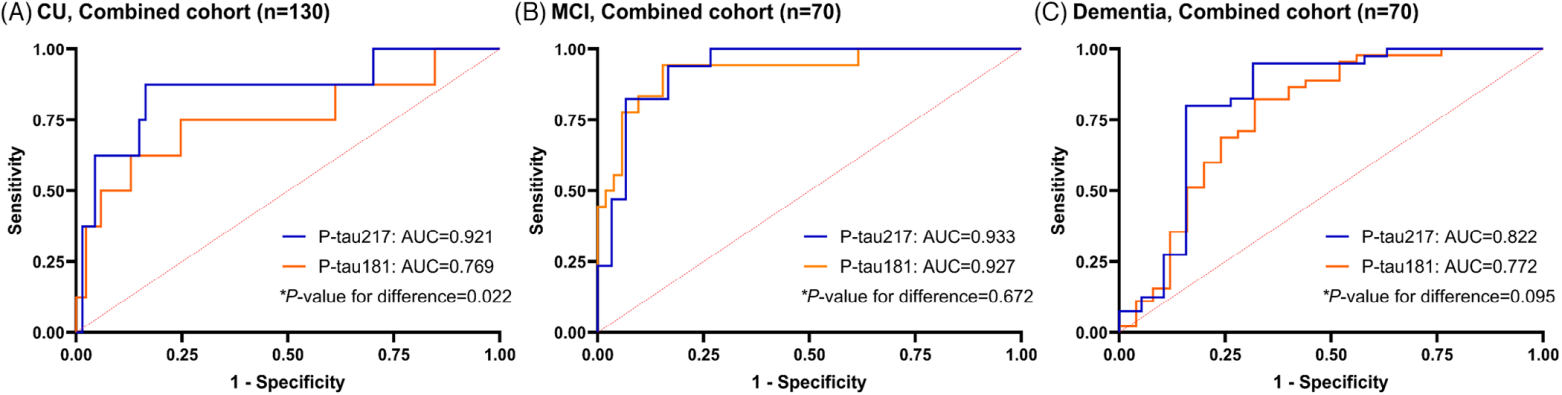
ROC curves for the ability of p‐tau217 and p‐tau181 to predict Aβ status at different cognitive stages. ROC curve analysis of p‐tau217 and p‐tau181 for predicting Aβ status at different cognitive stages. The ROC curves for p‐tau217 and p‐tau181 were compared between the CU (A), MCI (B), and dementia (C) groups. *AUCs of different ROC curves were compared using the DeLong method. Aβ, amyloid beta; AUC, area under the curve; CU, cognitively unimpaired; MCI, mild cognitive impairment; p‐tau, phosphorylated tau; ROC, receiver operating characteristic.

After adjusting for age and sex (Table 3 and Figure 2), plasma p‐tau217 (AUC = 0.936, 95% CI = 0.903–0.969) outperformed p‐tau181 (AUC = 0.877, 95% CI = 0.833–0.920) in predicting amyloid PET positivity (*P*_difference _< 0.001). The addition of *APOE* status (two‐biomarker model) to plasma p‐tau217 also outperformed the two‐biomarker model with p‐tau181 (*P*_difference _= 0.001, Figure 2). When the participants were divided according to cognitive ability, this difference was significant in the CU group (AUC of p‐tau217 = 0.921 vs. AUC of p‐tau181 = 0.769, *P*_difference _= 0.022; Figure 3A). In the MCI and dementia groups, there was no significant difference between p‐tau217 and p‐tau181 (*P*_difference _= 0.672 and 0.095, respectively; Figure 3B,C). However, in the dementia group, p‐tau217 tended to have better efficacy than p‐tau181 (AUC of p‐tau217 = 0.822 vs. AUC of p‐tau181 = 0.772, *P*_difference _= 0.095, Figure 3C), but the difference was not significant. In the combined cohort, the two‐biomarker model (including p‐tau [p‐tau217 or p‐tau181] and *APOE*) and the three‐biomarker model (including p‐tau, *APOE*, and GFAP) performed better than p‐tau alone but did not significantly improve the AUC (Table 3). The three‐biomarker model, including p‐tau217, *APOE*, and GFAP, had the highest discriminatory value (AUC = 0.946) and the lowest AIC (best model fit). These results were similar even when the Korean and Taiwanese cohorts were analyzed separately. The three‐biomarker model showed the highest discriminatory value in the Korean (AUC = 0.963) and Taiwanese (AUC = 0.968) cohorts. The two‐biomarker model with p‐tau217 and *APOE* demonstrated a high discriminative accuracy (AUC = 0.929 in the Korean cohort and AUC = 0.966 in the Taiwan cohort).

**TABLE 3:** Associations between amyloid PET positivity and plasma biomarker.

	Combined			Korean			Taiwanese		
Model	AUC (95% CI)	AIC (ΔAIC) vs. full model	*P* value vs. full model (vs. p‐tau217)^*^	AUC (95% CI)	AIC (ΔAIC) vs. full model	*P* value vs. full model (vs. p‐tau217)^*^	AUC (95% CI)	AIC (ΔAIC) vs. full model	*P* value vs. full model (vs. p‐tau217)^*^
1. p‐tau217, *APOE*, GFAP (three‐biomarker model)	0.946 (0.916–0.976)	146.3^a^, ^b^ (ref)	Ref (0.229)	0.936 (0.895–0.976)	94.3^a^, ^b^ (ref)	Ref (0.275)	0.968 (0.926–1.000)	57.1^a^, ^b^ (ref)	Ref (0.935)
2. p‐tau181, *APOE*, GFAP	0.926 (0.895–0.957)	189.9 (43.6)	0.163 (0.350)	0.924 (0.884–0.964)	116.3 (22.0)	0.122 (0.737)	0.947 (0.904–0.990)	75.8 (18.7)	0.072 (0.193)
3. p‐tau217, *APOE* (two‐biomarker model)	0.940 (0.909–0.972)	153.7 (7.4)	0.234 (0.540)	0.929 (0.886–0.973)	98.8 (4.5)	0.365 (0.448)	0.966 (0.923–1.000)	59.3 (2.2)	0.528 (0.931)
4. p‐tau181, *APOE*	0.895 (0.856–0.934)	222.4 (76.1)	0.001 (0.012)	0.884 (0.831–0.937)	145.2 (50.9)	0.007 (0.039)	0.925 (0.874–0.976)	79.4 (22.3)	0.024 (0.055)
5. p‐tau217	0.936 (0.903–0.969)	160.3 (14.0)	0.229 (ref)	0.922 (0.873–0.972)	99.4 (5.1)	0.275 (ref)	0.966 (0.922–1.000)	62.3 (5.2)	0.935 (ref)
6. p‐tau181	0.877 (0.833–0.920)	242.9 (96.6)	<0.001 (0.001)	0.886 (0.831–0.940)	153.2 (58.9)	0.022 (0.056)	0.846 (0.766–0.925)	89.8 (32.7)	0.004 (0.003)
7. GFAP	0.874 (0.828–0.919)	233.4 (87.1)	0.008 (0.002)	0.896 (0.841–0.951)	124.3 (30.0)	0.045 (0.177)	0.817 (0.723–0.910)	111.3 (54.2)	0.003 (0.001)
8. Age & sex	0.609 (0.531–0.687)	155.3 (9.0)	<0.001 (<0.001)	0.699 (0.611–0.786)	104.0 (9.7)	<0.001 (<0.001)	0.518 (0.390–0.647)	101.8 (44.7)	<0.001 (<0.001)

*Note*: Results from logistic regression models with amyloid positivity (cut‐off: Centiloid 37) as outcome. All models are adjusted for age and sex.

Abbreviations: AIC, Akaike information criterion; *APOE*, apolipoprotein E; AUC, area under the curve; CI, confidence interval; CU, cognitively unimpaired; GFAP, glial fibrillary acidic protein; MCI, mild cognitive impairment; NA, not applicable; PET, positron emission tomography; p‐tau, phosphorylated tau.

^a^ Best model fit.

^b^ Parsimonious model.

* *P* values are for comparisons of AUCs (using the DeLong test) to the full biomarker model.

### 3.3 Validating models from each cohort to other cohorts

**FIGURE 4:**
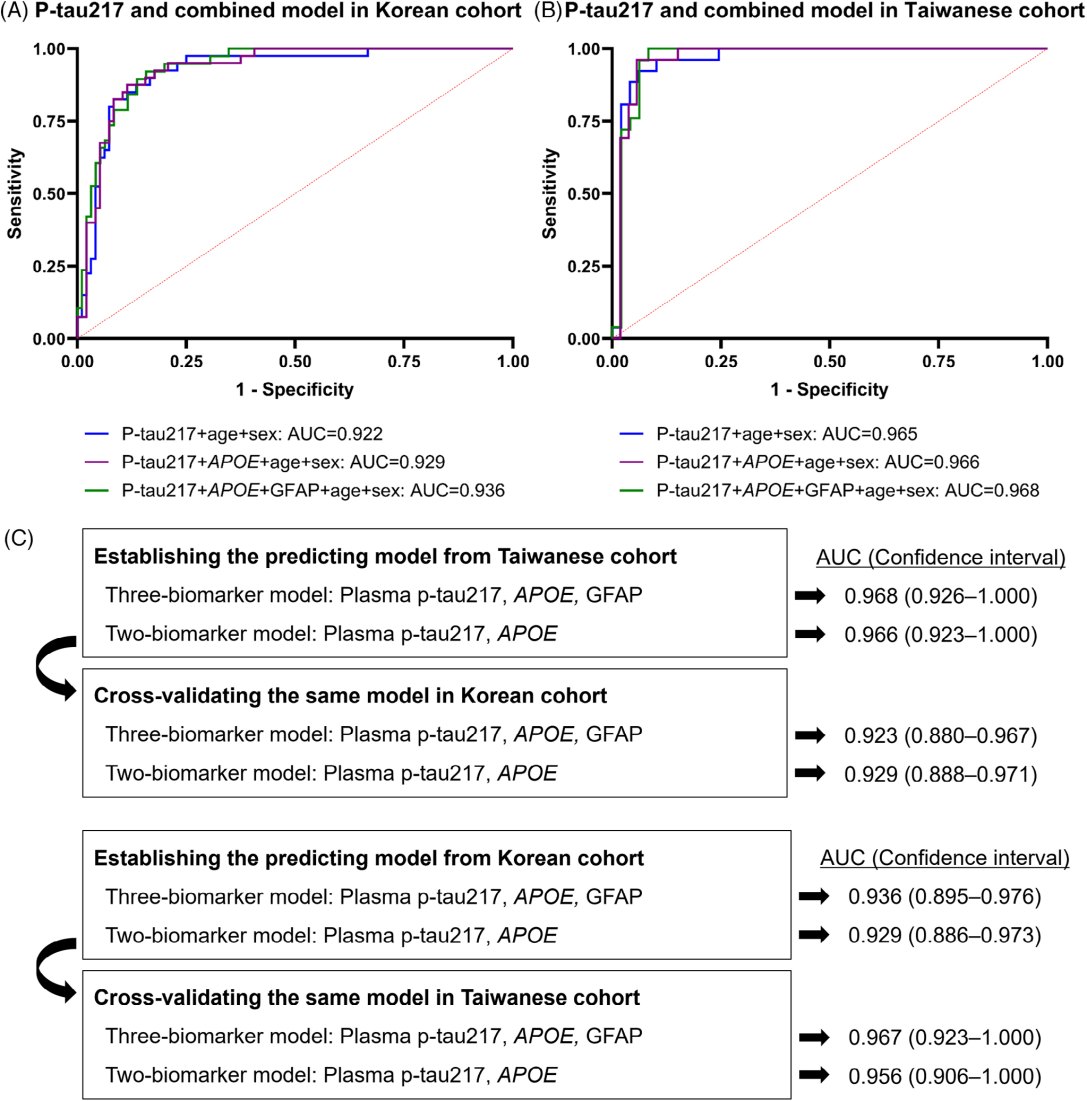
ROC curve for p‐tau217 for predicting Aβ status using PET in each cohort and the validation results. ROC curves are shown for plasma p‐tau217; a combination of plasma p‐tau217 and *APOE* status; and a combination of plasma p‐tau217, *APOE* status, and plasma GFAP in the Korean (A) and Taiwanese (B) cohorts. Logistic regression models established in the Taiwanese cohort were assessed in the Korean cohort, and the models established in the Korean cohort were assessed in the Taiwanese cohort (C). Aβ, amyloid beta; *APOE*, apolipoprotein E; AUC, area under the curve; GFAP, glial fibrillary acidic protein; PET, positron emission tomography; p‐tau, phosphorylated tau; ROC, receiver operating characteristic.

In both the Korean and Taiwanese cohorts, the three‐biomarker (p‐tau217, *APOE*, and GFAP) and two‐biomarker models (p‐tau217 and *APOE*) showed high discriminatory value in predicting amyloid PET positivity (Table 3, Figure 4A,B). The performance of these models was assessed in the other cohort (Figure 4C). When the models from the Taiwanese cohort were tested in the Korean cohort, both the three‐biomarker model (AUC = 0.923, 95% CI = 0.880–0.967) and the two‐biomarker model (AUC = 0.929, 95% CI = 0.888–0.971) showed high accuracy in predicting amyloid PET positivity. When the Korean models were tested in the Taiwanese cohort, both the three‐biomarker model (AUC = 0.967, 95% CI = 0.923–1.000) and the two‐biomarker model (AUC = 0.956, 95% CI = 0.906–1.000) also showed very high accuracy in predicting amyloid PET positivity.

### 3.4 Different thresholds to classify individuals into low‐, intermediate‐, and high‐risk groups

#### 3.4.1 Models based on p‐tau217 and combinations of additional covariates

**FIGURE 5:**
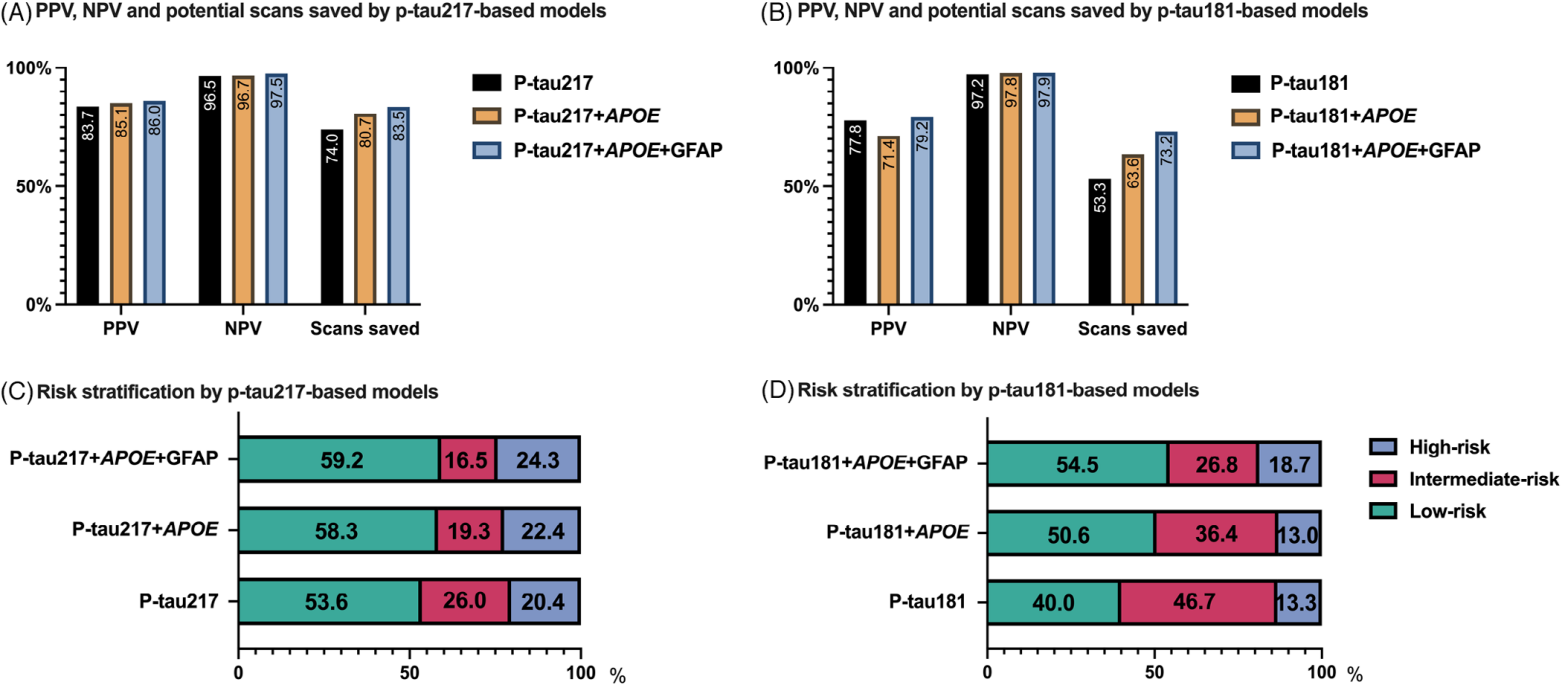
Risk stratification, PPV, NPV, and potential scans saved by different models. Panels (A) and (B) present the PPV (proportion of amyloid PET–positive cases in the high‐risk group), NPV (proportion of amyloid PET–negative cases in the low‐risk group), and potential proportion of scans saved (calculated as 100% minus the percentage of the intermediate‐risk group) across the different p‐tau217 and p‐tau181 models. The stacked bar charts display the proportion of each risk group across p‐tau217‐based models (C) and p‐tau181‐based models (D). All the models were adjusted for age and sex. *APOE*, apolipoprotein E; GFAP, glial fibrillary acidic protein; NPV, negative predictive value; PET, positron emission tomography; PPV, positive predictive value; p‐tau, phosphorylated tau.

When p‐tau217 was combined with age and sex (one‐biomarker model, AUC = 0.936, 95% CI = 0.903–0.969), the high sensitivity threshold (probability cutoff = 0.149) had an NPV of 96.5% (Figure 5A and Figure S1A in supporting information). The high specificity threshold (probability cutoff = 0.711) achieved a PPV of 83.7%. Through risk stratification, 20.4% of the participants were identified as high risk, 26% as intermediate risk, and 53.6% as low risk. The approach allowed for a potential reduction of 74% in PET scans (Figure 5B).

With the two‐biomarker model (*APOE* status, p‐tau217, age, and sex; AUC = 0.940, 95% CI = 0.909–0.972), the high sensitivity threshold (probability cutoff = 0.173) achieved an NPV of 96.7% (Figure S1B). The high specificity threshold (probability cutoff = 0.639) had a PPV of 85.1%. The model categorized 22.4% as high risk, 19.3% as intermediate risk, and 58.3% as low risk, potentially reducing 80.7% of PET scans.

With the three‐biomarker model (GFAP, p‐tau217, age, sex, and *APOE*, AUC = 0.946, 95% CI = 0.916–0.976), the high sensitivity threshold (probability cutoff = 0.204) achieved an NPV of 97.5% (Figure S1C). The high specificity threshold (probability cutoff = 0.549) had a PPV of 86.0%. Risk stratification included 24.3%, 16.5%, and 59.2% of participants in the high‐, intermediate‐, and low‐risk groups, respectively, significantly reducing the demand for PET scans by up to 83.5%.

#### 3.4.2 Models based on p‐tau181 and combinations of additional covariates

With the one‐biomarker model (p‐tau181, age, and sex; AUC = 0.877, 95% CI = 0.833–0.920), the high sensitivity threshold (probability cutoff = 0.134) had an NPV of 97.2% (Figure 5C and Figure S1D). The high specificity model (probability cutoff = 0.524) had a PPV of 77.8%. Risk stratification identified 13.3% of the participants as high risk, 46.7% as intermediate risk, and 40.0% as low risk, with a potential savings of 53.3% in PET scans (Figure 5D).

With the two‐biomarker threshold (*APOE* status, p‐tau181, age, and sex; AUC = 0.895, 95% CI = 0.856–0.934), the high sensitivity threshold (probability cutoff = 0.151) achieved an NPV of 97.8% (Figure S1E). The high specificity threshold (probability cutoff = 0.654) had a PPV of 71.4%. The risk classification for this model categorized 13.0% of the participants as high risk, 36.4% as intermediate risk, and 50.6% as low risk, potentially reducing 63.6% of PET scans.

With the three‐biomarker model (GFAP, p‐tau181, age, sex, and *APOE*, AUC = 0.926, 95% CI = 0.895–0.957), the high sensitivity threshold (probability cutoff = 0.143) achieved an NPV of 97.9% (Figure S1F). The high specificity threshold (probability cutoff = 0.569) had a PPV of 79.2%. Based on the model‐derived probabilities, the participants were classified as 18.7% (high risk), 26.8% (low risk), and 54.5% (intermediate risk), reducing the need for PET scans by 73.2%.

## 4 DISCUSSION

This study demonstrated that plasma p‐tau217 is a robust biomarker for predicting amyloid PET positivity, outperforming p‐tau181 in both the Korean and Taiwanese cohorts. Plasma p‐tau217 showed better discriminatory accuracy than p‐tau181, particularly in individuals who were CU. The combination of p‐tau217, *APOE* status, and GFAP further improved the model fit. The models developed in one cohort were confirmed in the other cohort, supporting the reliability of plasma p‐tau217 in different East Asian populations. Moreover, risk stratification using p‐tau217 significantly reduced the need for confirmatory amyloid PET imaging.

Significant differences in p‐tau217 (*P* value = 0.012), p‐tau181 (*P* value = 0.008), and GFAP (*P* value *= 0*.008) levels were observed between the Taiwanese and Korean cohorts (Table 1 and Figure S2A in supporting information). Comparing age‐, sex‐, cognitive stage‐, and amyloid‐positivity–matched participants (Figure S2B), there were no statistically significant differences in plasma p‐tau217 (*P* value = 0.080) or p‐tau181 (*P* value = 0.069) levels, although participants in the Korean cohort tended toward higher levels than those in the Taiwanese cohort. *Z* score transformations were applied to the biomarker data to minimize these differences and ensure comparability across cohorts. This variation could be attributed to ethnicity, genetic background, or the laboratory environment. Further studies, such as those analyzing the same samples in different laboratory environments or analyzing blood biomarkers in larger and more diverse populations, are needed to understand these discrepancies.

Multivariable logistic regression analysis revealed that plasma p‐tau217 and *APOE* ε4 carrier status were independent predictors of amyloid PET positivity. This finding aligns with previous studies in which p‐tau217 predicted amyloid positivity and that patients with AD frequently carry *APOE* ε4.^28^, ^29^ However, to our knowledge, this finding is unique as we adjusted for covariates including various blood biomarkers such as plasma p‐tau217, p‐tau181, GFAP levels, and *APOE* status. Unlike p‐tau217 and *APOE* ε4 carrier status, p‐tau181 and GFAP lost statistical significance after adjusting these covariates. These findings support the role of plasma p‐tau217 as a key component in non‐invasive diagnostic models of AD.

Interestingly, p‐tau217 performed better than p‐tau181 in predicting Aβ+, especially in CU participants. Previous studies have also reported that p‐tau217 performed better than p‐tau181 in detecting Aβ+.^7^, ^30^ Plasma p‐tau217 is known to increase longitudinally in preclinical AD, whereas no changes in p‐tau181 were noted.^31^ In the present study, a difference in the ability of p‐tau217 and p‐tau181 levels to predict Aβ+ was not detected in the MCI group, and the difference began to emerge again in patients with dementia, although it was not statistically significant (Figure 3). This finding suggests that p‐tau217 levels rise earlier than p‐tau181 levels and that the ability of p‐tau217 to detect AD might last longer.

We developed and confirmed several models using plasma p‐tau217 and p‐tau181 as key biomarkers for predicting amyloid PET positivity in patients with AD. These models integrated various biomarkers, including *APOE* ε4 status and GFAP, to increase the accuracy of the models for predicting amyloid pathology.

The first model incorporated p‐tau217 and p‐tau181 individually (one‐biomarker models), with adjustments for age and sex. In the combined cohort of Korean and Taiwanese participants, plasma p‐tau217 outperformed p‐tau181 in predicting amyloid positivity, with an AUC of 0.936 (95% CI: 0.903–0.969) for p‐tau217 compared to 0.877 (95% CI: 0.833–0.920) for p‐tau181. This pattern was observed across cognitive stages, especially in CU individuals, in which p‐tau217 performed significantly better (AUC = 0.921) than p‐tau181 (AUC = 0.769, *P*_difference _= 0.022). Although GFAP alone also showed a high discriminative value in predicting amyloid PET positivity in the combined cohort (AUC = 0.874, 95% CI = 0.828–0.919), it demonstrated significantly lower efficacy than p‐tau217. The model using only age and sex (AUC = 0.874, 95% CI = 0.828–0.919) did not show good performance (Table 3 and Figure S3 in supporting information).

We incorporated *APOE* ε4 status alongside plasma p‐tau to improve predictive accuracy, forming a two‐biomarker model. The addition of *APOE* status to p‐tau217 further increased the AUC to 0.940 (95% CI: 0.909–0.972) and notably increased the PPV (85.1%) in the high‐specificity model. These findings suggest that *APOE* status is essential for amyloid‐positive individuals. The two‐biomarker model with p‐tau217 and *APOE* outperformed the p‐tau181 version, highlighting the strength of p‐tau217 in predicting amyloid positivity.

The next step in model development involved adding GFAP to the existing two‐biomarker model to form a three‐biomarker model. This three‐biomarker model (p‐tau217, *APOE*, and GFAP) provided the best predictive accuracy, with an AUC of 0.946 (95% CI: 0.916–0.976) and the lowest AIC score, indicating the best model fit. The sensitivity and specificity were refined, with the high‐sensitivity model achieving an NPV of 97.5% and the high‐specificity model achieving the highest PPV of 86.0%.

We implemented a risk stratification approach based on the model‐derived probabilities of amyloid PET positivity, reducing the need for amyloid PET scans to only those classified as intermediate risk, which require priority confirmation through amyloid PET. The thresholds were set at 95% sensitivity (low risk) and 95% specificity (high risk). Regardless of the number of biomarkers included, p‐tau217–based models achieved an NPV of > 96% for low‐risk groups and a PPV > 83% for high‐risk groups, whereas 97% NPV and 71% PPV with p‐tau181–based models were achieved. The addition of more biomarkers reduced the proportion of individuals in the intermediate‐risk group, with the p‐tau217–based three‐biomarker model showing the lowest proportion (16.5%). In contrast, p‐tau181–based models showed a 10.3% to 20.7% increase in the intermediate‐risk group compared to p‐tau217 models. Nonetheless, longitudinal validation is crucial for confirming the reliability of these risk stratification models in reducing PET scan requirements while maintaining diagnostic accuracy.

Throughout model development, p‐tau217 consistently demonstrated superior performance compared to p‐tau181, particularly in CU participants. The integration of *APOE* and GFAP further enhanced the predictive value, making p‐tau217 the preferred biomarker for non‐invasively identifying amyloid pathology, especially in the early stages of AD.

To ensure the generalizability of the models, we conducted cross‐validation between the Korean and Taiwanese cohorts. Models developed in the Korean cohort were evaluated in the Taiwanese cohort and vice versa. The results confirm the high accuracy of the models across both populations.

Existing research, primarily in Western populations, underscores the utility of plasma biomarkers in predicting AD pathology. In the Mayo Clinic cohort,^32^ which included CU participants (*n* = 864), those with MCI (*n* = 148), and participants with AD dementia (*n* = 124), Quanterix plasma analytes including p‐tau181, Aβ42/Aβ40, GFAP, and neurofilament light chain, combined with age, sex, and *APOE*, achieved concordance (C) statistics of 0.78 to 0.82 for amyloid PET stages and 0.88 to 0.91 for intermediate/high versus none/low AD pathology. In the Lilly p‐tau217 subgroup, p‐tau217 outperformed p‐tau181 in distinguishing between the high and intermediate amyloid PET stages (C = 0.85). Another study, which combined three prospective cohorts from Sweden and Canada (total *n* = 1131),^33^ demonstrated that plasma p‐tau181 effectively distinguished AD dementia from Aβ– young adults (AUC = 0.994) and CU older adults (AUC = 0.902–0.982 across cohorts). In the primary care cohort of the same study, plasma p‐tau181 differentiated AD from young adults with high accuracy (AUC = 1) and CU older adults (AUC = 0.844), although it failed to distinguish AD from MCI (AUC = 0.55). A recent cross‐ethnic study conducted in Sweden (114 CU participants and 63 with MCI, 71 of whom had abnormal Aβ PET) and Japan (331 with CDR 0 and 143 with CDR 0.5, 81 of whom had abnormal Aβ PET)^34^ reported that plasma p‐tau 217 alone predicted abnormal Aβ PET with AUCs of 0.837 to 0.913. When combined with the Aβ42/Aβ40 ratio, *APOE* status, age, and sex, predictive performance improved to AUCs of 0.914 to 0.955. In the BioFINDER study, plasma p‐tau217 predicted AD progression within 4 years (AUC = 0.83), improving to 0.91 when combined with memory, executive function, and *APOE*. A similar result (AUC = 0.90) was observed for p‐tau181 in the ADNI cohort.^35^

Although Western cohorts have established the robust predictive value of plasma biomarkers, particularly when combined with clinical and genetic factors, such as *APOE*, data from East Asian populations remain limited. Our findings address this gap by demonstrating that p‐tau217 achieves high predictive accuracy, especially when combined with *APOE* and GFAP, across culturally and genetically distinct East Asian populations. This underscores the universal applicability of this biomarker and its potential for global clinical implementation in the early, non‐invasive detection of AD.

The high predictive value observed in CU participants highlights p‐tau217's potential to aid in early interventions, which are critical for advancing anti‐amyloid therapies. Additionally, our results confirm p‐tau217's reliability and versatility across East Asian populations. The use of two independent cohorts enhanced the generalizability of our findings, whereas cross‐validation between Korean and Taiwanese participants further reinforced the reliability of p‐tau217 as a predictive biomarker.

However, this study has some limitations. First, the commercial availability of the p‐tau217 and p‐tau181 assays is limited, which could hinder the broader application of this biomarker. Second, although our study focused on East Asian populations, further research is required to confirm whether our findings apply to other ethnic groups. Third, using model‐derived probabilities as cutoffs is not yet readily applicable in clinical practice, as it requires further standardization and accessibility for routine use. Finally, the participants in the current study were recruited from memory clinics in large hospitals. Therefore, replicating our findings in a larger population, including primary care settings, is necessary.

In conclusion, plasma p‐tau217 is a highly precise and non‐invasive biomarker for predicting amyloid pathology in patients with AD, offering a reliable alternative to amyloid PET imaging for early diagnosis and risk stratification, even in East Asian populations.

## AUTHOR CONTRIBUTIONS

Yung‐Shuan Lin, Hyuk Sung Kwon, Wei‐Ju Lee, Mina Hwang, Seong‐Ho Koh, Seong Hye Choi, and Jong‐Ling Fuh designed the study. Yung‐Shuan Lin and Hyuk Sung Kwon interpreted the data, generated the figures and drafted the manuscript. Seong‐Ho Koh, Seong Hye Choi, and Jong‐Ling Fuh supervised and revised the manuscript. Wei‐Ju Lee and Jee Hyang Jeong collected the data and supervised the study. Mina Hwang performed the experiments. All authors reviewed and approved the final version of the manuscript.

## CONFLICT OF INTEREST STATEMENT

The authors declare no conflicts of interest. Author disclosures are available in the supporting information.

## CONSENT STATEMENT

All of the participants provided written informed consent to participate in the study.

## Supporting information



Supporting Information

Supporting Information

## Data Availability

All data supporting this study will be shared with qualified academic researchers after obtaining consent to take part.
