# Supplementary material for: Cross‐cultural validation of plasma p‐tau217 and p‐tau181 as precision biomarkers for amyloid PET positivity: An East Asian study in Taiwan and Korea
Source: Alzheimers Dement. 2025 Jan 29;21(1):e14565. doi: 10.1002/alz.14565 (PMC11775528; doi:10.1002/alz.14565)
Supplement: Supplementary file 1 — Supporting Information [file ALZ-21-e14565-s001.docx]

**Supporting information**

**Cross-cultural validation of plasma p-tau217 and p-tau181 as precision biomarkers for amyloid PET positivity: An East Asian study in Taiwan and Korea**

Yung-Shuan Lin^a,b,c*^, Hyuk Sung Kwon^d*^, Wei-Ju Lee^c,e,f^, Mina Hwang^d^, Jee Hyang Jeong^g^, Seong-Ho Koh^d†^, Seong Hye Choi^h†^, Jong-Ling Fuh^a,b,c†^

^a^Department of Neurology, Neurological Institute, Taipei Veterans General Hospital, Taipei, Taiwan.

^b^School of Medicine, College of Medicine, National Yang Ming Chiao Tung University, Taipei, Taiwan

^c^Brain Research Center, National Yang Ming Chiao Tung University, Taipei, Taiwan

^d^Department of Neurology, Hanyang University Guri Hospital, Hanyang University College of Medicine, Guri, 11923, Republic of Korea

^e^Neurological Institute, Taichung Veterans General Hospital, Taichung, Taiwan

^f^Department of Post-Baccalaureate Medicine, College of Medicine, National Chung Hsing University

^g^Department of Neurology, Ewha Woman’s University School of Medicine, Seoul 07985, Republic of Korea

^h^Department of Neurology, Inha University College of Medicine, Incheon, 22332, Republic of Korea

**Equal Author Contribution:**

^*^ Yung-Shuan Lin and Hyuk Sung Kwon contributed equally to this work and should be considered co-first authors.

Seong-Ho Koh, Seong Hye Choi, and Jong-Ling Fuh are senior coauthors.

Table of contents

[Supplementary Table 1. Comparison of baseline characteristics between the Korean and Taiwanese cohorts. 3](#_Toc185980087)

[Supplement Figure 1. Probability distribution of each p-tau217 and p-tau181-based model in predicting amyloid positivity with a centiloid cutoff of 37. 5](#_Toc185980088)

[Supplement Figure 2. Differences in plasma biomarker levels between the Korean and Taiwanese cohorts. 6](#_Toc185980089)

[Supplement Figure 3. Amyloid prediction and risk stratification using GFAP, age, and sex 7](#_Toc185980090)

Supplementary Table 1. Comparison of baseline characteristics between the Korean and Taiwanese cohorts.

|  | All participants | | | Aβ-negative | | | Aβ-positive | | |
| --- | --- | --- | --- | --- | --- | --- | --- | --- | --- |
|  | Korean  (n = 170) | Taiwanese  (n = 100) | *P* | Korean  (n = 127) | Taiwanese  (n = 70) | *P* | Korean  (n = 43) | Taiwanese  (n = 30) | *P* |
| Demographics |  |  |  |  |  |  |  |  |  |
| Age, years | 69.9 ± 8.3 | 69.5 ± 8.5 | 0.736† | 68.4 ± 8.0 | 69.7 ± 8.0 | 0.293† | 74.0 ± 7.9 | 69.0 ± 9.8 | 0.018† |
| Sex, females (%) | 99 (58.2) | 46 (46.0) | 0.052 | 72 (56.7) | 32 (45.7) | 0.140 | 27 (62.8) | 14 (46.7) | 0.172 |
| Education, years | 9.5 ± 5.0 | 12.7 ± 3.9 | <0.001† | 9.9 ± 5.1 | 12.6 ± 4.0 | <0.001† | 8.3 ± 4.4 | 13.0 ± 3.7 | <0.001† |
| BMI, kg/m^2^ | 23.8 ± 3.0 | 24.1 ± 3.2 | 0.508† | 24.3 ± 3.0 | 24.4 ± 3.2 | 0.721† | 22.7 ± 2.6 | 23.4 ± 3.2 | 0.298† |
| Cognitive stage |  |  | 0.013 |  |  | 0.140 |  |  | 0.345 |
| CU (%) | 93 (54.7) | 37 (37.0) |  | 85 (66.9) | 35 (50.0) |  | 8 (18.6) | 2 (6.7) |  |
| MCI (%) | 41 (24.1) | 29 (29.0) |  | 31 (24.4) | 21 (30.0) |  | 10 (23.3) | 8 (26.7) |  |
| Dementia (%) | 36 (21.2) | 34 (34.0) |  | 11 (8.7) | 14 (20.0) |  | 25 (58.1) | 20 (66.7) |  |
| Medical History |  |  |  |  |  |  |  |  |  |
| Hypertension | 72/168 (42.9) | 36 (36.0) | 0.268 | 53/125 (41.7) | 27 (38.6) | 0.602 | 19 (44.2) | 9 (30.0) | 0.220 |
| Diabetes mellitus | 27 (15.9) | 14 (14.0) | 0.677 | 21 (16.5) | 12 (17.1) | 0.913 | 6 (14.0) | 2 (6.7) | 0.327 |
| Dyslipidemia | 62/167 (37.1) | 8 (8.0) | <0.001 | 52/124 (41.9) | 8 (11.4) | <0.001 | 10 (23.3) | 0 (0.0) | 0.004 |
| Coronary artery disease | 9 (5.3) | 5 (5.0) | 0.916 | 6 (4.7) | 4 (5.7) | 0.762 | 3 (7.0) | 1 (3.3) | 0.501 |
| Cerebrovascular disease | 7 (4.1) | 2 (2.0) | 0.349 | 5 (3.9) | 2 (2.9) | 0.695 | 2 (4.7) | 0 (0.0) | 0.231 |
| MMSE score, median (IQR) | 25.0 (21.0–28.0) | 26.0 (23.0–28.0) | 0.036‡ | 26.0 (22.0–28.0) | 27.0 (25.0–29.0) | <0.001‡ | 20.0 (15.0–25.0) | 21.5 (15.8–25.3) | 0.645‡ |
| CDR score, median | 0.0 (0.0–0.5) | 0.5 (0.0–0.5) | 0.005‡ | 0.0 (0.0–0.5) | 0.3 (0.0–0.5) | 0.002‡ | 0.5 (0.5–1.0) | 0.5 (0.5–1.0) | 0.092‡ |
| CDR-SOB score, median | 0.0 (0.0–1.5) | 1.0 (0.0–3.5) | 0.001‡ | 0.0 (0.0–0.5) | 0.3 (0.25–2.13) | 0.024‡ | 2.5 (0.5–5.0) | 3.3 (1.4–7.0) | 0.069‡ |
| APOE ε4 carrier | 45 (26.5) | 26 (26.0) | 0.932 | 24 (18.9) | 7 (10.0) | 0.101 | 21 (48.8) | 19 (63.3) | 0.221 |

NOTE. Data are presented as mean ± standard deviation, median (IQR) or number (%) unless otherwise indicated.

Cut-off for amyloid PET positivity: Centiloid 37. Pearson’s chi-squared test, †Student’s *t-*test and the ‡Mann–Whitney *U* test were used.

Abbreviations: PET, positron emission tomography; BMI, body mass index; CU, cognitively unimpaired; MCI, mild cognitive impairment; MMSE, Mini-Mental State Examination; IQR, interquartile range; CDR-SOB, Clinical Dementia Rating Scale sum of boxes.

# Supplement Figure 1. Probability distribution of each p-tau217 and p-tau181-based model in predicting amyloid positivity with a centiloid cutoff of 37.


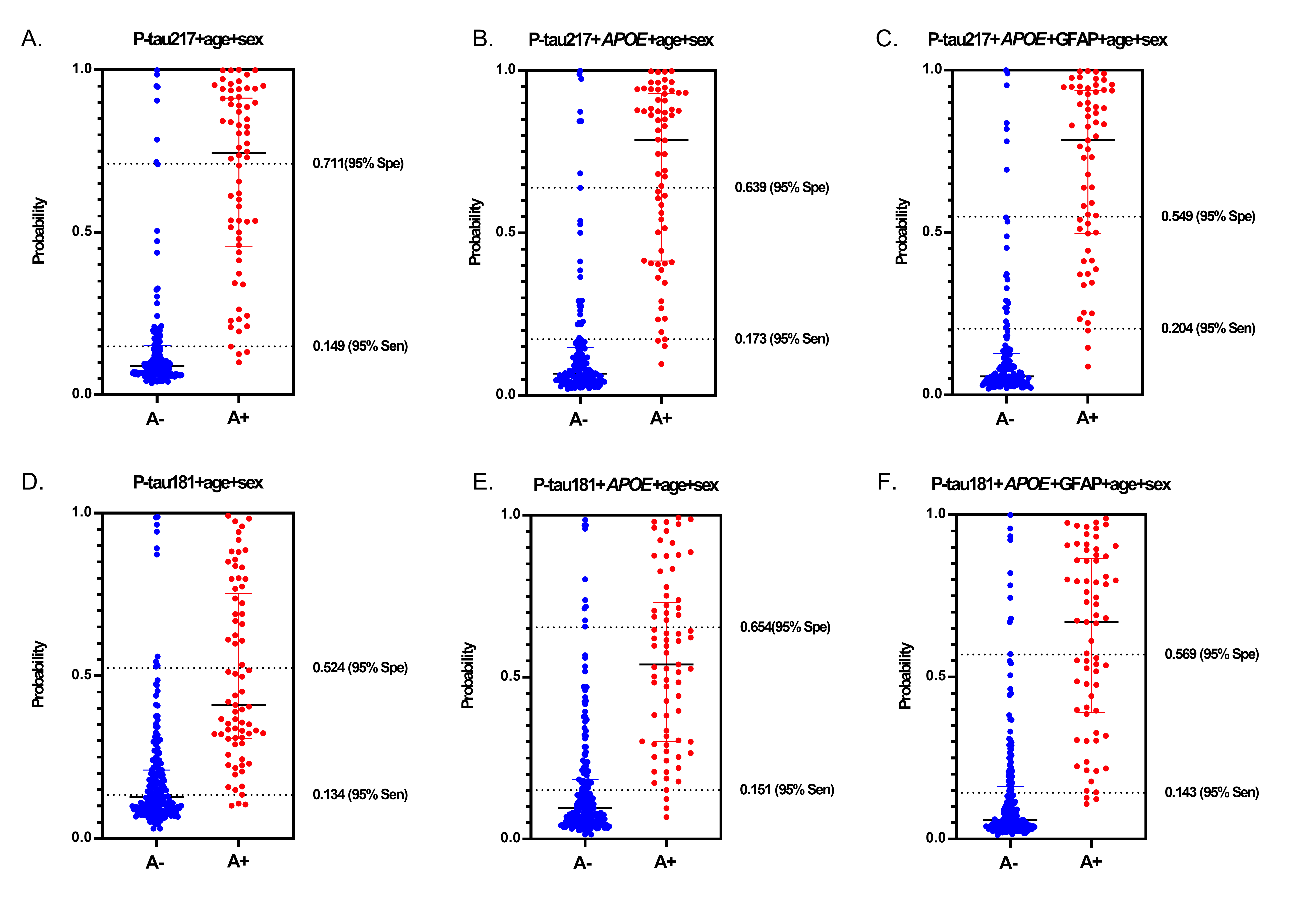


Panels A, B, and C represent p-tau217-based models incorporating one, two, and three biomarkers, respectively, while Panels D, E, and F show p-tau181-based models with the same biomarker combinations. All models are adjusted for age and sex. The dotted lines indicate the probability thresholds for achieving 95% sensitivity and 95% specificity.

Abbreviations: P-tau, phosphorylated tau; spe, specificity; sen, sensitivity; *APOE*, apolipoprotein E; GFAP, glial fibrillary acidic protein.

# Supplement Figure 2. Differences in plasma biomarker levels between the Korean and Taiwanese cohorts.


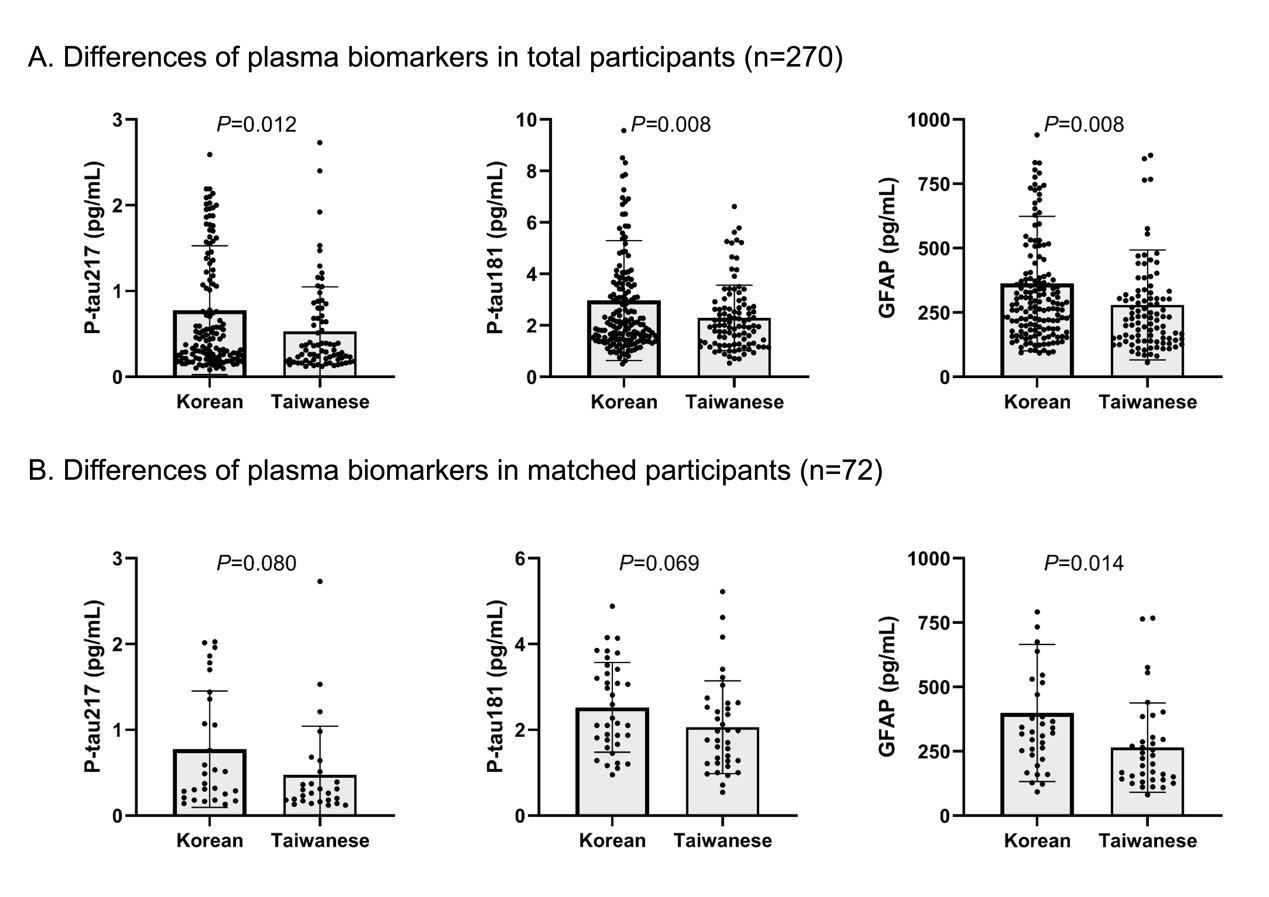


(A) In all participants, p-tau217 (*P* value=0.012), p-tau181 (*P* value =0.008), and GFAP (*P* value =0.008) levels were significantly higher in the Korean cohort compared to the Taiwanese cohort. (B) Among participants matched for age, sex, cognitive stage, and amyloid status, only GFAP levels remained significantly higher in the Korean cohort (*P* value =0.014).

# Supplement Figure 3. Amyloid prediction and risk stratification using GFAP, age, and sex

**
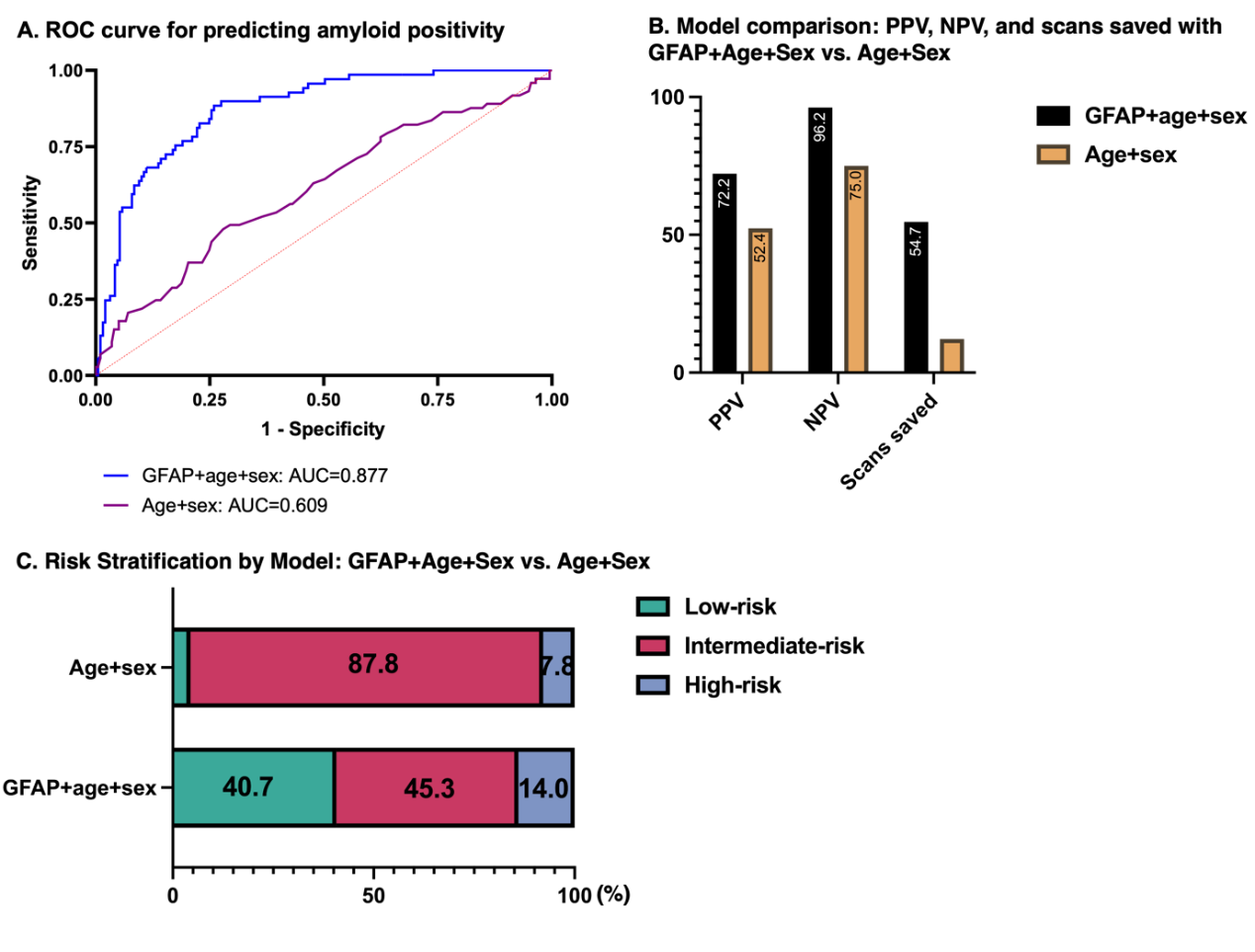
**

The figure illustrates a performance comparison between the age + sex and GFAP + age + sex models. Panel A displays the ROC curve and bar charts used to assess model performance. Panel B shows the positive predictive value, negative predictive value, and the percentage of scans potentially reduced by each model. Panel C depicts risk stratification across the two models.

Abbreviations: GFAP, glial fibrillary acidic protein; ROC, receiver operating characteristic; AUC, area under the curve; PPV, positive predictive value; NPV, negative predictive value.
